# The Establishment of Reference Intervals for Thyroid Hormone Tests in the Korean Population: Using a Direct Selection Technique

**DOI:** 10.3390/diagnostics15192510

**Published:** 2025-10-02

**Authors:** Jong Do Seo, Eun-Jung Cho, Changhee Ha, Hyung-Doo Park, Shinae Yu, Woochang Lee, Sollip Kim, Yeo-Min Yun

**Affiliations:** 1 Department of Laboratory Medicine, Konkuk University School of Medicine, Konkuk University Medical Center, Seoul 05030, Republic of Korea; 20210128@kuh.ac.kr (J.D.S.);; 2 Department of Laboratory Medicine, Hallym University Dongtan Sacred Heart Hospital, Hallym University College of Medicine, Hwaseong 18450, Republic of Korea; 3 Department of Laboratory Medicine and Genetics, Samsung Medical Center, Sungkyunkwan University School of Medicine, Seoul 06351, Republic of Korea; 4 Departments of Laboratory Medicine, Haeundae Paik Hospital, Inje University College of Medicine, Busan 48108, Republic of Korea; tlsdo55@naver.com; 5 Department of Laboratory Medicine, Asan Medical Center, University of Ulsan College of Medicine, Seoul 05505, Republic of Korealalacopine@gmail.com (S.K.)

**Keywords:** direct sampling technique, reference interval, thyroid function test, thyroid-stimulating hormone, thyroxine, triiodothyronine

## Abstract

**Background/Objectives**: Thyroid-stimulating hormone (TSH), free thyroxine (FT4), and total triiodothyronine (TT3) are biomarkers for evaluating thyroid function. Although hormone levels are affected by many biological and environmental factors, most laboratories use manufacturer-provided reference intervals (RIs) without considering these factors. Thus, in this study we assessed RIs for TSH, FT4, and TT3 in a Korean population, using a direct selection technique. **Methods**: Serum samples from patients without a history of thyroid disease, medication, family history, or antibody-positive test results were collected after a review of medical records. TSH, FT4, and TT3 levels were measured using the Cobas e801 analyzer (Roche Diagnostics GmbH, Mannheim, Germany) with dedicated reagents. RIs were then established using a non-parametric method, using values at the 2.5th and 97.5th percentiles as reference limits, which were then verified in a validation cohort. **Results**: A total of 618 subjects were enrolled in this study. Because the distribution of reference values for the four subgroups divided by sex and age (65 years) showed insignificant differences, combined RIs were determined, with the established RIs being 0.38–5.46 mIU/L for TSH, 12.28–22.40 pmol/L for FT4, and 0.94–2.32 nmol/L for TT3. When compared to manufacturer-claimed RIs, the Korean RI for TSH showed higher upper limits, while that for TT3 showed lower upper limits. Additionally, when newly established RIs were applied to the validation cohort, the rate of test-positive results decreased significantly. **Conclusions**: Significant differences in RIs for TSH and TT3 in the Korean population, compared to manufacturer-claimed values, highlight the need for population-specific RIs. Thus, interpreting the results for the Korean population requires caution, and Korean population-based RIs are necessary.

## 1. Introduction

As an endocrine organ, the thyroid gland produces and secretes hormones, such as thyroxine (T4) and triiodothyronine (T3), in response to the regulation of thyroid-stimulating hormone (TSH) secreted from the pituitary gland [1,2,3]. These hormones play a major role in coordinating normal organ functioning. For example, these hormones mediate normal development during infancy and regulate metabolic processes [3,4,5]. Among the hormones produced by systems in the hypothalamus–pituitary–thyroid axis that maintain homeostasis, TSH, free thyroxine (FT4), and total triiodothyronine (TT3) are regarded as important biological markers for assessing thyroid function, diagnosing diseases, and monitoring the response to therapy. Therefore, establishing a reliable standard for interpreting the measurement results of these analytes is essential [6,7].

Previous studies have shown that the concentration of these hormones is largely affected not only by biological factors, such as population, age, and sex, but also by environmental factors such as lifestyle and diet [8,9,10,11]. However, most commercial immunochemistry assays currently used in clinical laboratories rarely account for the influences of various factors when providing reference intervals (RIs) for analytes. Instead, many assays commonly present a single, global RI determined by their own criteria, including the thyroid hormone tests evaluated in this study. Information about the sampling technique for reference individuals, the statistical method, and characteristics of the reference population such as ethnicity, sex, or age are often treated as confidential and not disclosed to end users.

Thyroid hormone levels are affected by numerous factors. For example, in one study, the median TSH values across age groups showed the lowest value of 1.28 mIU/L in individuals in their 20s and the highest value of 2.08 mIU/L in those in their 80s, indicating a 1.6-fold difference [8]. Comparisons by race revealed a median value of 1.14 mIU/L in Black individuals and 1.43 mIU/L in Caucasians, reflecting a 1.2-fold difference [9]. As an environmental factor, high iodine intake, from food such as seaweed, was reported to transiently elevate TSH levels by up to 150% [10]. Therefore, using the RI derived from a reference group that has different characteristics from the clinical patient population in question may result in the misinterpretation of test results and lead to inappropriate medical decisions. For instance, the prevalence of subclinical hypothyroidism based on the National Health and Nutrition Examination Survey (NHANES) III in the United States was reported as 4.3% [12], whereas much higher rates were reported in Korean studies such as in the Ansung cohort (11.7%) and from the Korean Longitudinal Study on Health and Aging (KLoSHA, 17.3%) [13].

This discrepancy suggests that applying a global RI without considering population-specific factors may lead to overestimation of the prevalence of thyroid dysfunction. To address this, a previous study using the large-scale Korea National Health and Nutrition Examination Survey (KNHANES) dataset established thyroid hormone RIs for the Korean population [14]. However, that study reported a significantly higher TSH RI (0.62–6.86 mIU/L) compared to the manufacturer-claimed RI (0.27–4.20 mIU/L), which may not reflect clinical expectations and could complicate interpretation. In that study, an indirect sampling technique was applied, first by identifying the disease-free population using a self-reported questionnaire, followed by statistical exclusion of possible ‘unhealthy’ individuals from pre-existing data without access to comprehensive clinical information. To assess the validity of these findings and provide more clinically appropriate RIs for TSH, FT4, and TT3, the present study employed an a priori direct sampling approach, in which reference values were obtained through de novo measurements in reference individuals selected using predefined criteria. Reference individuals were collected for four subgroups divided by sex and age using a direct sampling technique. After establishing new RIs, the change in the proportion of test-positive results was investigated by applying the newly established RIs to a separate validation cohort.

## 2. Materials and Methods

### 2.1. Samples

Adult patients who visited the healthcare center or outpatient clinic at Konkuk University Medical Center or Hallym University Dongtan Sacred Heart Hospital between November and December 2023 underwent laboratory testing, and those with available residual serum samples were considered potential reference individuals. Then, the a priori direct sampling approach was adopted, in accordance with the Clinical and Laboratory Standards Institute (CLSI)’s EP28-A3c guideline [15], based on previously reported factors known to affect thyroid hormone levels. Patients who had (1) a prior diagnosis of, family history of, or current or past medication history for thyroid disease; (2) positive results for thyroid antibodies—anti-thyroglobulin (TG)—or anti-thyroid peroxidase (TPO); (3) the presence of non-thyroidal diseases such as hypertension, diabetes, cardiovascular disease, renal disease, chronic liver disease, or malignancy, or any current medication for these conditions; and (4) imaging findings suggestive of or consistent with the conditions above, were excluded after medical record review. In addition, patients visiting the obstetrics/gynecology clinic were excluded a priori to rule out female patients who were pregnant, and pregnancy status was verified through medical record review during the final selection process. In terms of sample factors, (1) samples suspected to contain endogenous interfering substances such as markedly elevated levels of hemoglobin, bilirubin, or lipids and (2) samples with insufficient residual volume to perform all the required measurements were also excluded. To avoid duplication, repeated blood sampling during the recruitment period were excluded based on institutional patient identification numbers and only the first available residual serum of each subject was used. To ensure analyte stability, fresh serum samples stored and transported at 4 °C and analyzed within seven days of collection were used.

### 2.2. Assay

Measurements of three thyroid hormones—TSH, FT4, and TT3— for obtaining reference values and two antibodies—anti-TG and anti-TPO—for applying exclusion criteria were performed on the study samples collected. Because variations in test results can occur depending on the assay method used—even for the same sample [5,16,17,18,19]—all measurements in this study were performed using the Cobas 8000 e801 analyzer (Roche Diagnostics GmbH, Mannheim, Germany) and its dedicated reagents, Elecsys TSH, Elecsys FT4 III, and Elecsys T3 (Roche Diagnostics), which are routinely used at our institution. Each assay was traceable to the 2nd IRP WHO Reference Standard 80/558, Enzymun-Test which was standardized using equilibrium dialysis, and reference standards by weighing T3 into an analyte-free human serum matrix, respectively.

### 2.3. Statistical Analysis

In accordance with the CLSI EP-28A guidelines [15], the nonparametric method was used to determine the reference intervals, defined as the values between the 2.5th and 97.5th percentiles of each subgroup. The necessity of RI partitioning by sex and age was evaluated using the Harris–Boyd test [20]. After establishing the RIs, a validation cohort was constructed using laboratory data from patients who visited the Konkuk University Medical Center between January and June 2024 and who underwent thyroid hormone testing. The newly established RIs were then applied to evaluate changes in the proportion of test-positive results.

Statistical analyses were conducted using Microsoft Excel 2019 (Microsoft, Redmond, WA, USA), IBM SPSS Statistics version 29.0 (IBM Corp., New York, NY, USA), and MedCalc version 14.8.1 (MedCalc Software Ltd., Ostend, Belgium).

## 3. Results

**Figure 1:**
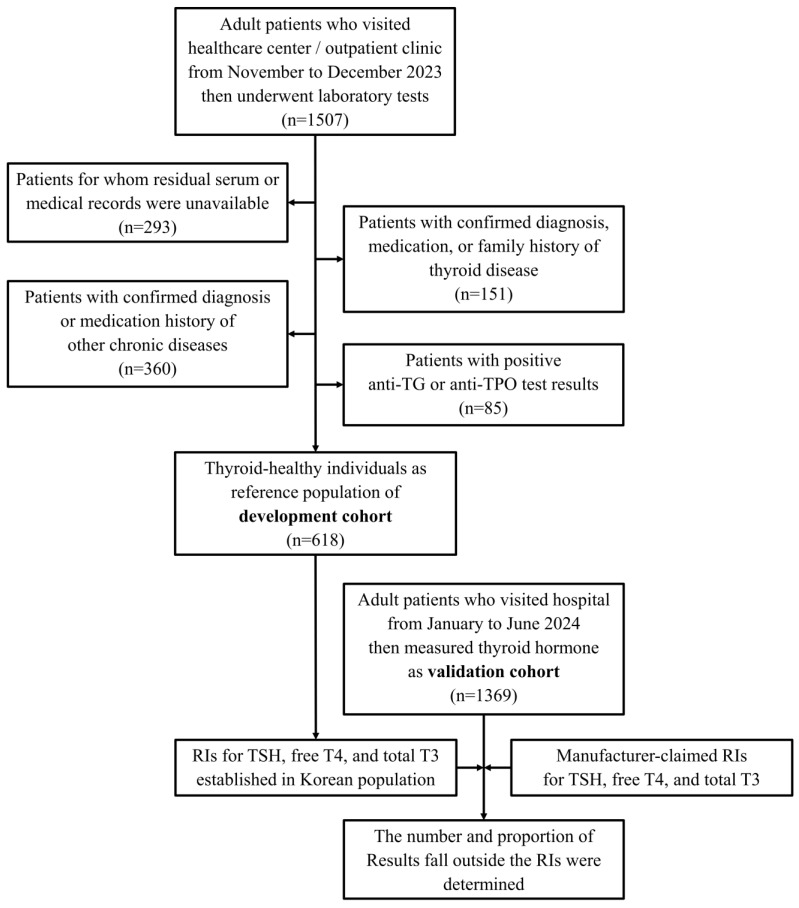
Flow of the study. The RIs for thyroid hormones were established by reference values obtained from development cohort; then, the changes in the number and proportion of results falling outside the RIs in validation cohort were investigated.

A total of 618 reference individuals were included in the final analysis after medical record review: 185 men aged 20–65 years (young male), 141 men aged ≥65 years (older male), 149 women aged 20–65 years (young female), and 143 women aged ≥65 years (older female) (Figure 1). Two TSH measurements falling outside the assay’s analytical measurement range were excluded from the RI calculation.

The RIs of TSH in each subgroup were 0.645–4.90 mIU/L for young males, 0.355–6.21 mIU/L for older males, 0.339–6.15 mIU/L for young females, and 0.159–6.23 mIU/L for older females (Table 1). For FT4, the RIs were 13.64–24.04, 10.93–22.14, 12.25–20.47, and 12.06–23.17 pmol/L for the respective subgroups. For TT3, the RIs were 1.15–2.37, 0.66–2.49, 1.16–2.26, and 0.88–2.30 nmol/L, respectively.

**Figure 2:**
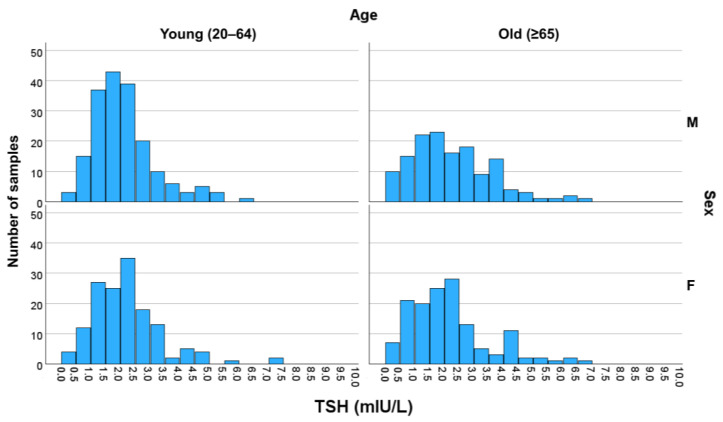
Distribution of TSH value in each subgroup. The number of samples with TSH levels divided by a range of 0.5 mIU/L intervals for each subgroup is presented in the histogram above. Three samples with values higher than 10.0 mIU/L were omitted for readability. Higher distributions than the manufacturer-claimed RIs were observed in all subgroups. Abbreviations: F, female; M, male; TSH, thyroid-stimulating hormone.

When compared with the manufacturer-claimed Ris—TSH, 0.270–4.20 mIU/L (reported in µIU/mL, conventional unit); FT4, 11.97–21.88 pmol/L (0.93–1.70 ng/dL); and TT3, 1.23–3.07 nmol/L (0.80–2.0 ng/mL)—a notable upward shift in the upper reference limit of TSH was observed in all subgroups (Figure 2), with the lower bound of the 90% confidence interval (CI) exceeding the claimed upper limit of 4.20 mIU/L for all the subgroups. For TT3, the newly derived RIs demonstrated a downward shift. The lower reference limit and its 90% CI upper bound were lower than the claimed lower limit (1.23 nmol/L) in all subgroups except that for young males, and the upper reference limit and its 90% CI upper bound were below the claimed upper limit (3.07 nmol/L) in all but the older male subgroup. By contrast, the FT4 RIs did not show statistically significant differences from the claimed RIs.

**Figure 3:**
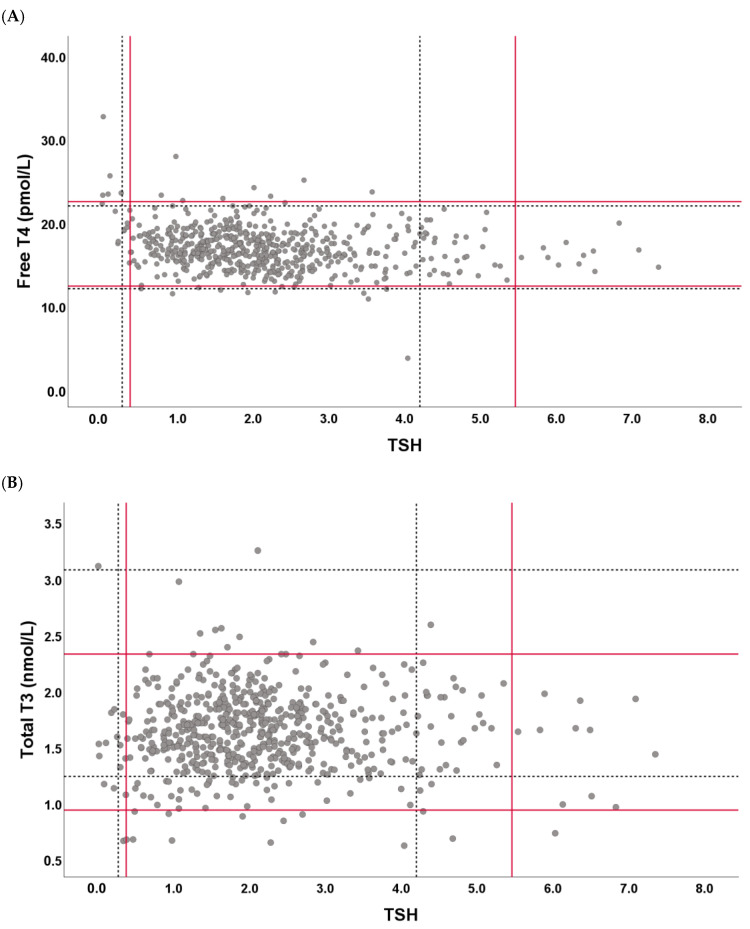
TSH, FT4, and TT3 reference values and established/manufacturer-claimed reference intervals. In the scatter plot above, each dot represents the reference values for TSH, (**A**) FT4, and (**B**) TT3, with the dotted line indicating the manufacturer-claimed reference limits and the solid red line indicating the reference limits established in this study. The shift in the upper reference limits for TSH and lower upper limits for TT3 is visualized.

The Harris–Boyd test was applied to assess whether RI partitioning by age and sex was statistically justified. No significant differences were found across the four subgroups, indicating that a combined RI derived from the entire reference population was acceptable. The RIs derived from the whole reference group for thyroid hormones were 0.375–5.46 mIU/L for TSH, 12.23–22.40 pmol/L for FT4, and 0.94–2.32 nmol/L for TT3. Compared to the claimed RIs, the same upward shift for TSH and downward shift for TT3 were observed in the combined population, as seen in individual subgroups (Figure 3).

The claimed RIs and the new RIs established in this study were applied to a validation cohort consisting of adult patients who visited our hospital between January and June 2024 and underwent thyroid hormone tests. Excluding the case of repeated tests in the same individual, a total of 1369 patients were recruited as the validation cohort. Of the 1369 patients, 1136 underwent all three tests, 174 underwent TSH and FT4 tests, 58 only underwent the TSH test, and 1 only underwent the TT3 test (Table 2). The number and proportion of results falling outside the claimed and newly established RIs were 19.6% (268/1368) versus 16.2% (222/1368) for the TSH test and 13.3% (151/1137) versus 6.3% (72/1137) for TT3, respectively, resulting in a significant difference. For FT4, which did not show a significant difference between claimed and established RIs, the rates of results falling outside each RI were equal to 13.2% (173/1310).

Focusing on the clinical impact of the TSH upper limit and TT3 lower limit changes, the proportion of patients classified with subclinical hypothyroidism—defined as elevated TSH with normal FT4—significantly decreased from 8.0% (105/1310) based on the claimed RI to 3.3% (43/1310) with the newly established RI. Similarly, the proportion of patients falling below the TT3 lower limit, a criterion for low T3 syndrome, decreased from 12.7% (144/1137) to 3.1% (35/1137).

## 4. Discussion

The establishment of reference intervals (RIs) is essential for interpreting clinical laboratory results and guiding medical decisions. However, RIs based on reference populations that differ demographically from the target clinical population may compromise diagnostic accuracy. In commercial immunoassay systems, manufacturer-provided RIs are widely used, yet the process of deriving them—including reference population characteristics and statistical methodology—is often undisclosed. This underscores the importance of establishing population-specific RIs that better reflect local clinical contexts.

In this study, the RIs for TSH and TT3 established from a Korean reference population significantly differed from the manufacturer-provided values. These discrepancies likely stem from ethnic, demographic, and environmental differences between populations. By contrast, FT4 levels showed no significant deviation, indicating relative consistency across populations for this marker.

The upward shift in the upper reference limit for TSH observed here aligns with prior Korean studies using both the same [14,19,21] and different [19,22] analytical systems to ours. However, the RI derived in this study for TSH (0.38–5.46 mIU/L) was lower than those from the study employing indirect sampling on KNHANES data (0.62–6.86 mIU/L), likely due to the stricter clinical criteria applied in our a priori, direct sampling approach. Prior research has linked elevated TSH levels in Koreans to high dietary iodine intake, supported by urinary iodine measurements [14] and dietary questionnaires [22].

These findings reinforce concerns that applying values derived from non-Korean reference populations may lead to an overestimation of test-positive findings in Koreans. For instance, TSH and FT4 tests are most commonly used to screen for thyroid dysfunction, and elevated TSH levels are commonly regarded as signs of suspicious hypothyroidism. However, the distribution of TSH levels in Koreans is higher than that of claimed RIs; therefore, existing RIs may cause overestimation of the prevalence of hypothyroidism by increased false-positive decisions. This is consistent with a study performed as part of the KNHANES [14]. Here, an increase in TSH levels in a population with high iodine intake was confirmed through a review of past studies, and high iodine intake in the Korean population was demonstrated via urine iodine measurement. Notably, the 2023 Clinical Practice Guidelines of the Korean Thyroid Association recommend a higher TSH reference limit of 6.8 mIU/L [23] based on KNHANES data, distinct from the 4.0 mIU/L suggested by the European Thyroid Association [24], which was adopted from manufacturer-claimed values. This represents a unique RI for the Korean population.

The RIs established in our study provide a population-specific estimate that reflects the known upward shift in TSH levels observed in the Korean population. The authors of previous RI studies using KNHANES data applied an indirect sampling technique, selecting a presumed disease-free population based on self-reported questionnaires and applying statistical methods to estimate the RI without access to detailed clinical information. While such an approach is practical when large datasets are available, the lack of clinical validation may lead to biased RI estimates, depending on the distribution of the source population. In fact, past studies demonstrated that indirect sampling methods applied to the same dataset could yield significantly different RI estimates compared to those obtained by direct sampling, highlighting potential limitations and inconsistencies in indirect approaches [25,26].

By contrast, the a priori direct sampling method used in our study applied predefined clinical criteria based on medical records, which enhances the reliability of the reference population and minimizes the risk of including ‘unhealthy’ individuals. Although our RI for TSH was slightly lower than that reported in studies using the indirect method with KNHANES data, it still confirms the upward shift relative to manufacturer-claimed values and reflects the iodine-rich dietary environment in Korea. Importantly, this approach allows for a more transparent and clinically interpretable RI that may reduce ambiguity in diagnosis and treatment decisions, particularly in cases of borderline thyroid dysfunction.

Several limitations should be considered. First, the direct sampling approach does not include a statistical outlier-removal step; it relies on excluding “unhealthy” individuals by medical record review. Because this exclusion depended on the completeness of records available at our institutions, we cannot rule out incomplete removal of individuals with disqualifying conditions. Second, although each subgroup met the CLSI EP28-A3c minimum (*n* ≥ 120) for nonparametric RI estimation, subgroup sample sizes were still limited and therefore underpowered to detect subtle differences by sex or age reported in other studies [5,8,10,11]. In calculating the 90% CIs for the reference limits, the observed minimum and maximum reference values had a strong influence on the CI bounds. While combining subgroups according to the Harris–Boyd test increased the sample size, potential outliers that were not fully excluded may still affect CI estimation for specific subgroups. Moreover, even after combining subgroups, the overall number of reference individuals remained modest, which likely contributed to wider CIs for the reference limits; additionally, the right-skewed distribution of TSH makes the upper limit particularly susceptible to variability and can give the impression of an expanded RI. Third, although we confirmed differences between the RIs determined in this study and those provided by the manufacturer, the causes of these differences could not be investigated because detailed information on reference individuals was limited. For instance, iodine nutrition was not assessed in our study, and this limitation—despite the fact that study population was unlikely to differ materially from previous Korean cohorts demonstrating high dietary iodine intake [14,21]—may limit the interpretation of the upward shift observed in TSH. Similarly, clinical information was limited for subjects in the validation cohort; consequently, while we found reductions in the proportion of test-positive results for TSH and TT3, with a marked decrease in patients classified as having subclinical hypothyroidism (8.0% to 3.3%) and low-T3 syndrome (12.7% to 3.1%) (Table 2), we were unable to systematically assess concordance with definitive clinical diagnoses. Fourth, the RIs established here were derived using assays from a single manufacturer (Roche), and global standardization/harmonization of thyroid hormone measurements remains incomplete [19]. Therefore, direct transferability of these RIs to other analytical platforms is limited. While RIs may be adoptable when the comparability of an analytical system and subject population are confirmed [15], such an approach requires rigorous comparison of methods. Therefore, it is advisable to consider establishing manufacturer-specific Korean RIs, especially when significant inter-assay variability is observed [19,23]. Last, reference samples were obtained from two geographically proximate institutions, which may limit the representativeness of our reference population for the entire Korean population. Future studies may require a broader sampling strategy to improve generalizability.

Despite these limitations, this study established RIs for thyroid hormones using a priori direct sampling in a reference population that had the same characteristics as clinical patients; thus, it is expected to be a useful tool for the interpretation of test results, such as screening and diagnosing disease, as well as monitoring response to therapy. The results of this study can be verified by comparing them with the results of our ongoing study, in which we adopted an indirect sampling method based on medical record data.

## 5. Conclusions

Thyroid hormone levels are influenced by various demographic and environmental factors, and reference intervals (RIs) derived from the Korean National Health and Nutrition Examination Survey (KNHANES) using an indirect sampling method showed substantial differences from global RIs. Since RI estimation is affected by the selection of reference individuals, we conducted a study using a direct sampling approach based on clinical information to evaluate its impact. Our findings confirmed previously observed shifts in the reference limits via indirect methods, emphasizing the importance of applying RIs derived from clinically homogeneous populations for accurate interpretation.

## Figures and Tables

**Table 1:** Reference intervals of the serum TSH, FT4, and T3 levels established in this study and manufacturer-claimed reference intervals.

	*n*	2.5th Percentile (90% CI)	Median (90% CI)	97.5th Percentile (90% CI)
TSH (mIU/L)				
M (age 20–64)	185	0.645 (0.297–0.787)	1.90 (1.82–2.05)	4.90 (4.52–6.36)
F (age 20–64)	149	0.339 (0.109–0.686)	2.09 (1.82–2.29)	6.15 (4.51–11.3)
M (age ≥ 65)	140	0.355 (0.007–0.426)	2.01 (1.82–2.23)	6.21 (4.81–18.6)
F (age ≥ 65)	142	0.159 (0.014–0.525)	1.94 (1.70–2.15)	6.23 (4.66–29.9)
Combined	616	0.375 (0.257–0.495)	1.99 (1.87–2.06)	5.46 (4.82–6.36)
Manufacturer-claimed	516	0.270		4.20
FT4 (pmol/L)				
M (age 20–64)	185	13.64 (11.91–14.16)	17.25 (16.99–17.51)	23.04 (21.62–24.97)
F (age 20–64)	149	12.23 (11.43–13.13)	16.22 (15.83–16.48)	20.47 (19.44–25.49)
M (age ≥ 65)	141	10.94 (3.64–12.47)	16.73 (16.35–17.25)	22.14 (21.37–71.31)
F (age ≥ 65)	143	12.10 (4.97–13.00)	16.22 (15.83–16.60)	23.17 (20.59–32.57)
Combined	618	12.23 (11.58–12.87)	16.60 (16.48–16.86)	22.40 (21.62–23.43)
Manufacturer-claimed	801	11.97		21.88
TT3 (nmol/L)				
M (age 20–64)	185	1.15 (0.975–1.24)	1.72 (1.67–1.75)	2.37 (2.23–2.96)
F (age 20–64)	149	1.15 (1.01–1.22)	1.64 (1.58–1.69)	2.26 (2.09–2.43)
M (age ≥ 65)	141	0.660 (0.613–0.874)	1.58 (1.52–1.63)	2.49 (2.20–3.24)
F (age ≥ 65)	143	0.891 (0.723–1.06)	1.54 (1.48–1.61)	2.30 (2.14–2.58)
Combined	618	0.937 (0.766–1.01)	1.63 (1.60–1.66)	2.32 (2.23–2.43)
Manufacturer-claimed	514	1.23		3.07

Abbreviations: CI, confidence interval; F, female; FT4, free thyroxine; M, male; TSH, thyroid-stimulating hormone; TT3, total triiodothyronine.

**Table 2:** Comparison of test-positive result rates and clinical classifications using claimed vs. newly established reference intervals.

Analyte or Clinical Condition	*n*	Claimed RI, *n* (%)	New RI, *n* (%)
TSH	1368	268 (19.6%)	222 (16.2%)
FT4	1310	173 (13.2%)	173 (13.2%)
Subclinical hypothyroidism (high TSH/normal FT4)	1310	105 (8.0%)	43 (3.3%)
TT3	1137	151 (13.3%)	72 (6.3%)
Low T3 syndrome (TT3 below reference lower limit)	1137	144 (12.7%)	35 (3.1%)

Comparison of test-positive rates and clinical classifications using claimed versus newly established reference intervals (RIs). The number and proportion of patients falling outside the claimed and newly established RIs for each analyte (TSH, FT4, and TT3) and clinical conditions are shown.

## Data Availability

The datasets generated during and/or analyzed during this study are not publicly available due to restrictions imposed by the institutional review board (IRB), which does not permit the transfer of raw data outside the research institution.
